# Bioanalytic Hybrid System Merging 3-Dimensional Cell Culture and Chromatographic Precision for Unprecedented Preclinical Insights in Molecular Imaging

**DOI:** 10.2967/jnumed.124.269133

**Published:** 2025-05

**Authors:** Verena Pichler, Verena Schwingenschlögl-Maisetschläger, Irem Duman, Xavier Monforte, Stefanie Ponti, Lukas Zimmermann, Elma Joldic, Monika Dumanic, Chrysoula Vraka, Marcus Hacker, Christian Kraule, Andreas Herbert Teuschl-Woller

**Affiliations:** 1 Department of Pharmaceutical Chemistry, University of Vienna, Vienna, Austria;; 2 Department of Biomedical-Imaging and Image-Guided Therapy, Medical University of Vienna, Vienna, Austria;; 3 PhaNuSpo Doctoral School, University of Vienna, Vienna, Austria;; 4 Department of Life Science Engineering, University of Applied Sciences Technikum Wien, Vienna, Austria;; 5 Division of Medical Radiation Physics, Department of Radiation Oncology, Medical University of Vienna, Vienna, Austria; and; 6 DOC medikus GmbH, Krems, Austria

**Keywords:** tissue engineering, radiotracer development, artificial extracellular matrix

## Abstract

We introduce a unique bioanalytic hybrid system for the preclinical assessment of radiotracer candidates, combining a 3-dimensional cell culture on an artificial extracellular matrix functioning as a stationary phase and a chromatographiclike system array. **Methods:** Silk fibroin sponges were applied to simulate an extracellular matrix and to function as a stationary phase. Different cell lines were grown on the silk scaffold and used to investigate radiopharmaceuticals in a small-animal PET/CT system. **Results:** Our system integrates with the strength of chromatographic systems, allowing high throughput, full automation, and online processing with the complexity of an advanced 3-dimensional cell culture for in vitro modeling of real tissuelike geometry, morphology, and dynamics. **Conclusion:** This system holds great potential to study newly developed radiotracers for applications in binding studies and assessment of unspecific binding. It might help to decrease the translational gap from in vitro cell cultures to in vivo studies while it aligns with the 3R principle (reduce, replace, refine) of animal testing.

## Visual Abstract

**Figure:**
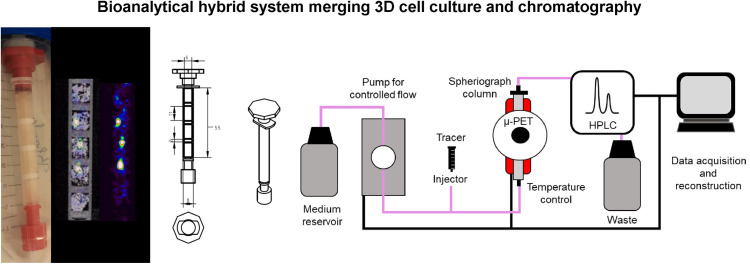

Molecular imaging using radiotracers for SPECT or PET has revolutionized both fundamental research and the medicinal world by visualizing physiologic and pathophysiologic processes. The exponentially growing interest in specialized radiopharmaceuticals worldwide is strongly coupled to the Food and Drug Administration approval of the theranostic radiopharmaceuticals Pluvicto (^177^Lu-vipivotide tetraxetan; Novartis) and Lutathera (^177^Lu-DOTATATE; Novartis) (1). Preclinical evaluation of radiopharmaceuticals differs from regular drug development. For imaging probes, no side effects are expected from the tracer amounts applied (a nano- to picomolar mass), but the total-body distribution must be considered to evaluate the target-to-background noise ratio for high-quality imaging. In particular, imaging quality is affected by nondisplaceable and off-target binding. Knowledge of the tracers’ biodistribution, and a thorough preclinical evaluation, are crucial for high-resolution diagnostics and targeted therapy with minimal side effects. The gold standard is still animal experiments, with the usual translational problems or lack of sufficient cellular or mouse models. The limitation in reliably predictive and high-throughput methodology for evaluating novel radiotracer candidates in a human cell- and tissue-based preclinical testing scheme has motivated the development of the hybrid, dynamic column-based 3-dimensional (3D) cell culture system described in this paper.

## MATERIALS AND METHODS

We strongly aimed to implement the Food and Drug Administration’s Critical Path Initiative and the 3Rs (reduce, replace, refine) of animal testing (2). We combined the principles of chromatographic systems with advanced concepts from tissue engineering. This involved constructing a biocompatible silk sponge–based stationary phase to serve as an artificial extracellular matrix or tissue analog and to immobilize mammalian cells. These cells were well nourished through a continuous flow of cell culture medium as the mobile phase. So far, only a limited number of dynamic 3D cell cultures have been described for PET applications and for evaluation or reevaluation of a set of radiotracer candidates (3,4).

**FIGURE 1.**
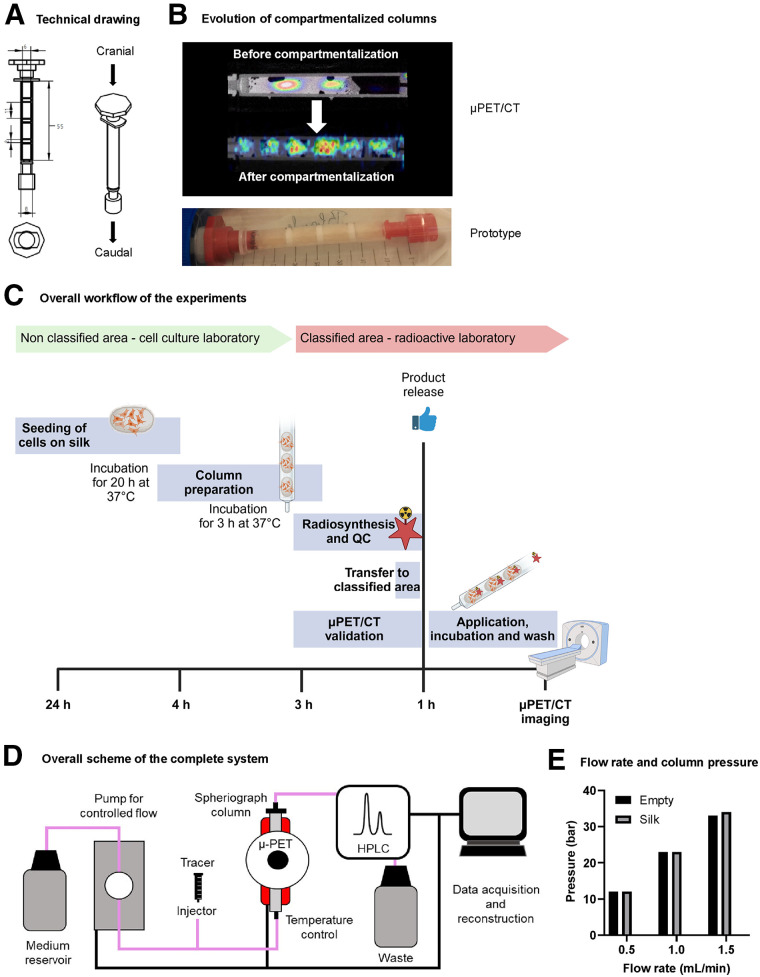
Technical setup of silk sponge as stationary phase and column body. (A) Technical drawing of column body including flow direction (cranial to caudal). (B) Stepwise improvement by changing from noncompartmentalized to compartmentalized system. From top to bottom: final composition of column including silk, frits, and caps; PET/CT image of preliminary setup without compartments; CT image of final setup including compartments separated by frits. (C) Logistic setup of column preparation, radiotracer production, transfer to radioactive area, and measurement within 24-h time frame (created in bioRender). (D) Setup for automated administration of radiotracer to column and schematic representation of overall system. (E) Pressure limitation test of system for empty and filled columns. No additional pressure was introduced in system using silk scaffolds. HPLC = high-performance liquid chromatography; QC = quality control.

Silk sponges, a well-established substrate for cell growth, have a unique physicochemical and morphologic cross-linked structure that allows for both high structural stability and high integrity under dynamic flow conditions (Fig. 1A) (5). Additionally, silk scaffolds are ideal for our purpose as they show high stability toward radiation (6) and have been tested extensively for short- and long-term application in in vitro culture (7). These features allow the application of any adherend cell line onto the silk sponge, whereby the column shape enables minimal void volumes resulting in reduced radioactive waste. The primary design consisted of the column body, silk sponges including the cell lines of interest, and the mobile phase. However, we extended the system to a compartmentalized technical setup by introducing frits between the sponge cutouts. These frits reduce cellular migration; facilitate small-animal PET image postprocessing; and enable the harmonization, standardization, and reproducibility of the results (Fig. 1B; Supplemental Figure 1–2; supplemental materials are available at http://jnm.snmjournals.org). Fast and reliable results require a precise logistic setup because of time limitations caused by decay of the investigated radioisotopes, including ^11^C, ^18^F, and ^68^Ga. This setup includes parallel handling of column and cell preparation, radiotracer production and quality control, a standardized protocol for radiotracer application and validation, and scanning of the system with small-animal PET (Fig. 1C). The used materials must be compatible and allow assessment with a combination of CT and MRI. A major part of the development process involved optimizing the required equilibration volume and time, flow conditions, and detection limits to ensure optimal fluidic conditions, minimize artifacts, prevent leakages, and achieve conditions with limited shear stress on cells. This helped maintain the integrity of results unless shear stress was intentionally controlled or set (Figs. 1D–1E) (8). The overall time required to seed the cells on the scaffolds and prepare the columns is comparable to that of conventional 2-dimensional (2D) cell culture. The processing time for the radioactive steps—application, incubation, and washing—was automated with 2 different pump systems (Supplemental Fig. 3). This automation can significantly reduce both the handling time and the radiation dose. Introduction of an investigational radiotracer allows simultaneous assessment of receptor affinity and saturation, nondisplaceable binding to the silk-based cell carrier acting as a tissue analog, and selectivity to the target. The cellular biochemistry is monitored by small-animal PET/CT but can be expanded to other imaging modalities such as optical imaging and MRI.

## RESULTS

**FIGURE 2.**
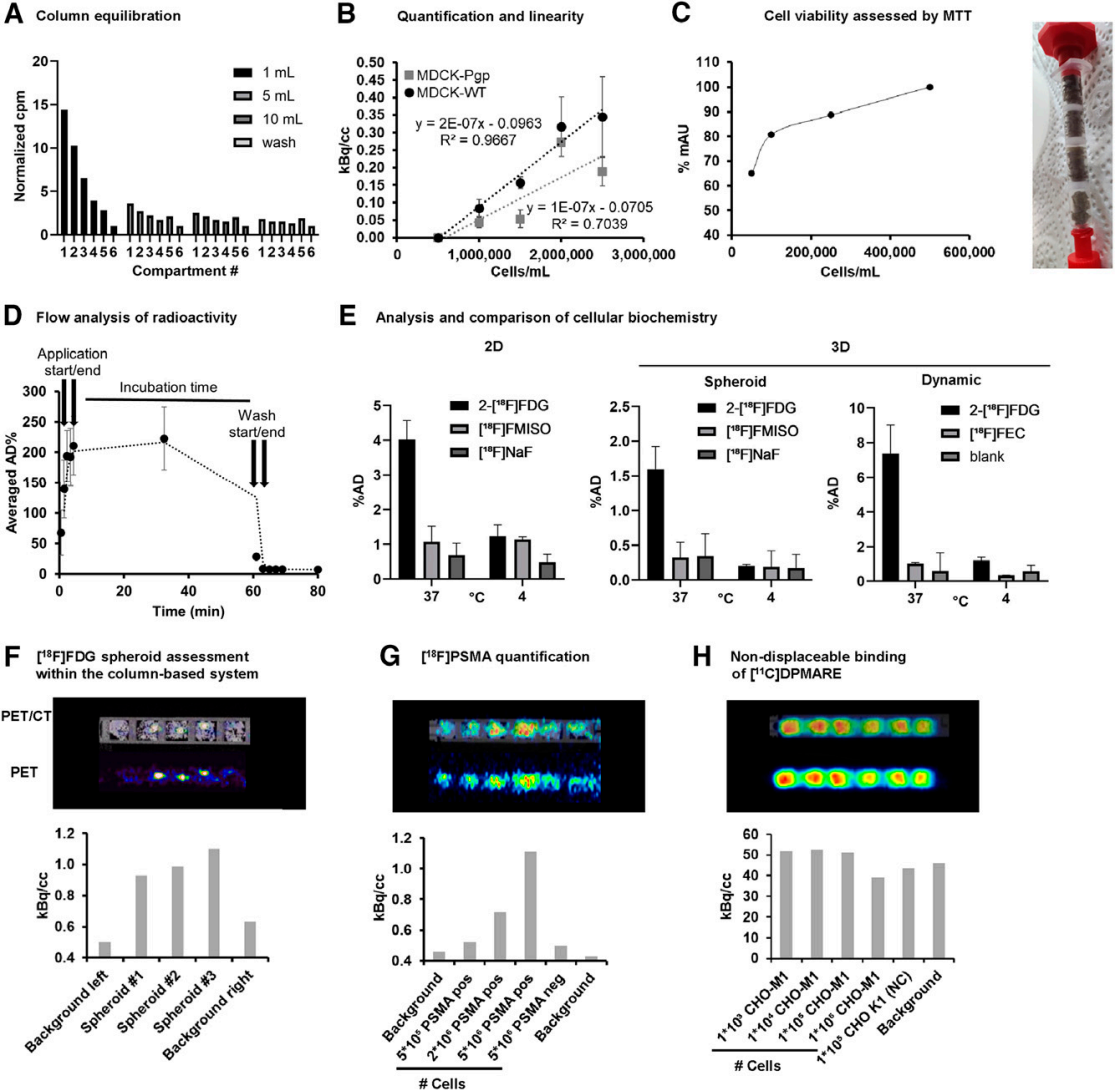
Radiotracer interaction studies in dynamic 3D cell culture system. (A) Analysis of void volume and administration volume of system to ensure equal medium distribution for incubation. (B) Quantification of cellular accumulation of radiotracer [^18^F]FDG as marker for cell viability and proliferation in MDCK-WT and MDCK-Pgp cells (*n* = 3). Cell line–dependent accumulation was observed, with higher reproducibility for MDCK-WT than for MDCK-Pgp cells. (C) Cell viability assessment of seeded cells in dependency on cell count to estimate ideal cell seeding capacity to avoid overgrowth in HCT116 cells after 5 d (*n* > 3), and MTT (3-[4,5-dimethylthiazol-2-yl]-2,5 diphenyl tetrazolium bromide) assay performed to analyze cell viability within column. (D) Dynamic scan to analyze fluidic behavior of administered radiotracer. (E) Comparison of behavior of HT29 cells in monolayer, multicellular tumor spheroid, and our dynamic system exposed to [^18^F]FDG (active transport), [^18^F]fluoromisonidazole (passive distribution), [^18^F]fluoroethylcholine (active transport), and [^18^F]NaF (passive distribution) at 37°C and 4°C. (F) Small-animal PET/CT scan of 600-μm-sized HT1080 spheroids incubated with [^18^F]FDG (*n* = 4, 1 representative dataset is shown to visualize direct readout), and analysis of cell viability by [^18^F]FDG. (G) Quantification of PET tracer with high specific binding and low nondisplaceable binding. Radiotracer interaction of [^18^F]FPSMA was investigated on PC3-PSMA–positive cells (increasing cell number in 5 × 10^4^ to 5 × 10^6^ cells per sponge) in comparison to PC3-PSMA–negative cells and silk without cells as background (*n* = 3, 1 representative dataset is shown). (H) Quantification of investigational PET tracer with high specific binding and high nondisplaceable binding. [^11^C]DPMARE (investigational radiotracer toward muscarinic acetylcholine receptors) was investigated in CHO-M1, expressing human M1 subtype of muscarinic acetylcholine receptors, and CHO-K1 cells as negative control (*n* = 2, 1 representative dataset is shown). AD = applied dose; FEC = fluoroethylcholine; FMISO = fluoromisonidazole.

The choice of the correct plastic type for the column body was an integral part to avoid image artifacts based on nonspecific binding of the radiotracers to the synthetic polymers. Evaluation of the binding behavior of a set of radiotracers ranging from low to high lipophilicity revealed polyethylene terephthalate as the plastic type of choice (Supplemental Fig. 4). Column and method validation involved estimation of the column’s extra column (void volume) and equilibration volume at a flow of 1 mL/min (9). The column’s void volume was found to be low, at 0.25 mL, with a total calculated column volume of 1.55 mL (Fig. 2A).

The column equilibration process was tested by introducing up to 20 mL of [^18^F]FDG, and the distribution of the radiotracer was followed over 20 min. There was an invasion phase of around 1 mL, and total equilibration was found after 10 mL (equal radioactivity distribution throughout the column), equal to around 10 column void volumes. This equilibration volume of 10–20 column volumes is also recommended for analytic chromatographic systems. Consequently, we set all required fluid transfer processes—medium introduction, washing or removal processes—to 10 mL. Figure 2D shows the dynamic flow behavior of the introduced radioactivity of [^18^F]FDG in the composite system without cells over the experiment’s course of time, including an equilibration phase with 10 mL (flow rate, 1 mL/min), a 1-h incubation time (stopped flow), a 10-mL washing phase (flow rate, 1 mL/min), and remaining radioactivity at the end of the experiment. The introduction of cells allowed evaluation of cell viability, molecular functions, and long-term survival within our system in comparison to conventional 2D and 3D cultures. Cellular uptake of [^18^F]FDG was found to depend on cell line and cell number, proving that the cells are viable and have an active metabolism (Fig. 2B). Cell viability was studied over 5 d with an increasing initial cell number of 50,000–500,000/mL. Cells remained viable throughout the entire period, but reduced growth was observed after 5 d in the compartments with an initial concentration of 250,000 and 500,000 cells/mL. This reduction in growth was attributed to contact inhibition, which occurs when cell growth is restricted because of limited space (Fig. 2C) (10). The system was further validated in comparison to a conventional monolayer (2D) and a multicellular tumor spheroid (3D) (spheroid characterization is described in Supplemental Fig. 5) and evaluated regarding cellular uptake and interaction for [^18^F]FDG, [^18^F]fluoromisonidazole, [^18^F]fluoroethylcholine, and [^18^F]NaF in HCT116, HT1080, and HT29 cells at 37°C and 4°C. Cold exposure attenuated the active transport of [^18^F]FDG and [^18^F]fluoroethylcholine to background levels (Fig. 2E; Supplemental Figs. 6 and 7). For metabolic tracers, similar results were observed for all 3 methods (2D, spheroid, and column-based; Supplemental Fig. 8) and cell lines. [^18^F]FDG showed the highest accumulation (active glucose metabolism), followed by [^18^F]fluoroethylcholine (active cell proliferation), and—as negative controls—[^18^F]fluoromisonidazole (potential hypoxic regions) and [^18^F]NaF (no active transport) exhibited background levels.

## DISCUSSION

The validation showed encouraging similar cellular behavior in the established models (2D and 3D spheroid culture) versus our new model, with an absence of hypoxic or malnourished regions. However, our dynamic system allowed assessment of additional parameters such as nondisplaceable binding. In the final step, more complex studies were conducted involving various applications including the evaluation of multicellular tumor spheroids introduced in the column, receptor interaction, and quantification of binding affinities. The introduction of 600-μm multicellular tumor spheroids of HT1080 cells (Fig. 2F; Supplemental Fig. 9) demonstrated the high sensitivity of our system as evidenced by the similar cellular uptake of [^18^F]FDG within the spheroid. Our system’s versatility and potential were evident in binding experiments with different radiotracers, such as [^18^F]FPSMA-1007 and the investigational compound [^11^C]DPMARE (radiotracer candidate targeting muscarinic acetylcholine receptors) (11). Using highly lipophilic neuroradiotracers, our system measures receptor interaction in real time and additionally depicts potential off-binding to cells without the receptor and nonspecific binding to the silk scaffold mimicking extracellular matrix. The model clearly identified radiotracers with the potential for in vivo application, showing a receptor-dependent signal (Fig. 2G) while also distinguishing radiotracers with insufficient binding capacity, as indicated by receptor-independent signals (Fig. 2H).

## CONCLUSION

We introduce a unique and universal bioanalytic system that merges a chromatographiclike setup with 3D cell cultures. This system enables the rapid and automated analysis of cellular behavior by applying well-established radiotracers and facilitates high-throughput screening of the binding capacity of radiotracer candidates. This high-throughput dynamic method might thereby reduce the radiation dose to personnel in medical imaging. The controlled application of radiotracers at defined speeds ensures the even distribution of radiation doses and essential nutrients, CO_2_, and O_2_ to viable cells. Our proof-of-concept study shows that the method is versatile and simple and has the potential to accelerate and make radiotracer development more accessible.

## DISCLOSURE

This work was supported by the FFG Bridge 30 (Spheriograph 877136). Christian Kraule owns DocMedikus GmbH and holds patent P3546563 A1 20191002. No other potential conflict of interest relevant to this article was reported.

## Notes

KEY POINTS **QUESTION:** Can we close the gap between in vitro and in vivo investigations to expedite the radiotracer development process? **PERTINENT FINDINGS:** The establishment of a robust, dynamic 3D cell culture system that combines the resilience of a chromatographic system with the efficiency of advanced tissue engineering enables high-throughput screening of radiotracer candidates. This approach allows for innovative screening of radiotracers without the need for animal testing, with its ethical considerations. **IMPLICATIONS FOR PATIENT CARE:** Accelerating the process of radiotracer screening and development enables the rapid translation of more innovative radiotracers into clinical use.
